# *GmRWP-RK1* Enhances Salt Tolerance by Modulating Antioxidant Defense, Ion Homeostasis and Stress-Responsive Pathways in Soybean

**DOI:** 10.3390/plants15060912

**Published:** 2026-03-16

**Authors:** Lu Liu, Qianyue Bai, Min Xu, Qi Zhang, Yuhong Gai, Naveed Ahmad, Piwu Wang, Zhuo Zhang, Nooral Amin, Wei Jian

**Affiliations:** 1 Plant Biotechnology Centre, College of Agronomy, Jilin Agricultural University, Changchun 130118, China; 2 Institute for Safflower Industry Research of Shihezi University, Pharmacy College of Shihezi University, Key Laboratory of Xinjiang Phytomedicine Resource and Utilization, Ministry of Education, Shihezi 832003, China

**Keywords:** *GmRWP-RK1*, cloning, Arabidopsis, salt stress, RT-qPCR, hairy roots, enzymatic activities, soybean

## Abstract

Soil salinity is rapidly spreading across agricultural regions and has become one of the most critical constraints on soybean growth, yield, and sustainable production. Despite the central role of transcription factors (TFs) in coordinating plant responses to abiotic stresses, the molecular mechanisms by which *RWP-RK* domain-containing TFs regulate salt-tolerant responses in soybean remain poorly understood. Our previous genome-wide characterization identified 28 *RWP-RK* TFs in soybean exhibiting abiotic stress-responsive expression, yet their biological functions under salt stress have not been experimentally validated. Here, we investigated a 981-bp *GmRWP-RK1* encoding region and demonstrated its regulatory role in enhancing salt tolerance by activating antioxidant defence, Na^+^/K^+^ homeostasis, and transcriptional control of salt-responsive genes using a cross-species overexpression approach. The two Arabidopsis lines (OE1 & OE4) overexpressing *GmRWP-RK1* demonstrated significantly improved salt tolerance, as evidenced by ~18% greater survival and enhanced germination compared to non-transgenic plants under salinity stress. This phenotype was supported by stronger antioxidant protection, as indicated by elevated proline levels, reduced MDA accumulation, and increased SOD and POD activities. At the molecular level, the transgenic lines also showed up-regulated expression of key stress-responsive genes (*AtACS10*, *AtSUMO1*, *AtGBF1*), confirming the regulatory influence of *GmRWP-RK1* on salt-adaptation pathways. Consistent with the Arabidopsis results, *GmRWP-RK1* overexpression in soybean hairy roots also led to improved salt-stress tolerance by accumulating significantly reduced ROS contents (27.38% lower H_2_O_2_ and 33.98% lower O_2_^−^), and maintained a balanced Na^+^/K^+^ ratio compared to that of non-transgenic hairy roots under salinity. Furthermore, *GmRWP-RK1*-overexpressing transgenic soybean hairy roots showed increased expression of stress-responsive genes, especially *GmATG-5*, *GmOLP-1*, and *GmOLP-2*. Overall, our results support a possible role of *GmRWP-RK1* in soybean salt tolerance and provide a foundation for future functional and breeding-oriented studies.

## 1. Introduction

Soybeans are a nutritional powerhouse, offering a rich source of protein, healthy fats, essential micronutrients, and bioactive compounds that contribute significantly to human and livestock health. Beyond their nutritional value, soybeans are integral to a wide range of industrial and sustainable agricultural applications, including protein-rich animal feed, soil fertility improvement, crop rotation practices, biofuels, soy-based plastics, and industrial lubricants [1]. Through symbiotic nitrogen fixation, soybean cultivation reduces the need for chemical fertilizers, contributing to environmentally friendly farming systems [2]. Although soybean is one of the most widely cultivated legume crops globally, with annual production exceeding 350 million tons, soybean production is frequently threatened by abiotic stresses such as drought, salinity, and temperature extremes, which significantly reduce yield and quality [3,4]. Among these, soil salinity is increasingly recognized as a major limitation to sustainable soybean production worldwide, underscoring the urgent need to develop salt-resilient cultivars.

Transcription factor (TF) families constitute key regulators of plant responses to environmental stresses and represent promising targets for engineering stress-resilient crops [5]. Major TF families studied for improving stress tolerance include *MYB* [6], *MADS-BOX* [7], *WRKY* [8], *HD-Zip*, *bZIP* [9,10], and *ARF* [11]. Recently, the *RWP-RK* TF family has emerged as an important regulator of stress responses in plants [12,13,14]. *RWP-RK* genes regulate key growth and developmental processes and are ubiquitous in plants, contributing to adaptation to external stresses. Prior studies on the plant *RWP-RK* family suggest that they play key roles in nitrogen responses and gametophyte development [15]. Similarly, comparative analyses have reported roles for *RWP-RK* genes in regulating nitrogen signaling and nodulation responses in Arabidopsis and legume crops [16]. The promoter regions of *RKD* genes are enriched in MeJA and ABA-responsive elements, which could enhance their responsiveness to abiotic stresses [14,17]. Researchers have also suggested that *GmRWP-RK1* and *GmRWP-RK2* regulate nodule formation by binding to the promoters of *GmYUC2*, *GmSPL9*, and *GmNIN* [18]. In our previous genome-wide study, we also identified 28 *RWP-RK* genes and revealed their differential expression under various abiotic stress conditions [18]. Given the broader regulatory functions of *RWP-RKs* across plant species, their potential roles in coordinating salt-stress tolerance in soybean remain largely unexplored, particularly for individual members such as *GmRWP-RK1*.

Although *RWP-RK* transcription factors have been classically associated with nitrogen signaling, nitrate assimilation, and reproductive development, accumulating evidence suggests that they also contribute to abiotic stress responses. For example, overexpression of an *RWP-RK* gene in pearl millet enhanced heat tolerance, accompanied by increased antioxidant enzyme activities (SOD and POD) and reduced MDA levels, suggesting mitigation of oxidative damage [19]. Recent studies have shown that *RWP-RK* genes are involved in responses to heat and salt stress, indicating stress-inducible expression [14,18]. Moreover, *NLPs* respond to various abiotic stresses in rice and Arabidopsis; a notable increase in the expression of *AtNLP4* and *AtNLP9* was observed under heat stress [15]. The role of the *OsNLP4-OsMADS27* module in governing nitrate-dependent salt tolerance in rice has been investigated [20]. Additionally, *AtNLP* and *RKD* are involved in gametophyte growth and regulating physiological and metabolic processes during drought and heat stress [13,14,21]. These findings provide important insights into the possible role of *RWP-RK* TFs in enhancing stress adaptation beyond growth and developmental responses. Together, emerging evidence supports the view that *RWP-RK* TFs can integrate nutrients and stress cues. However, the specific downstream targets and partner proteins are likely differ between *NLP* and *RKD*-type regulators, as well as among species. However, despite their stress-responsive expression, functional evidence for *RWP-RK*-mediated salt tolerance in soybean, particularly for RKD-related candidates such as *GmRWP-RK1*, remains limited. In this study, we aimed to elucidate how *GmRWP-RK1* (Glyma. 01G159200) contributes to salt-stress tolerance by focusing on the physiological, biochemical and molecular responses of transgenic plants. Using cross-species overexpression in Arabidopsis and soybean, we investigated whether *GmRWP-RK1* positively regulates salt-tolerant responses by maintaining photosynthetic potential and reducing ROS accumulation (H_2_O_2_^−^ and O_2_^−^), strengthening antioxidant enzyme activities (SOD, POD, and CAT), improving osmoprotectant and damage indicators (proline and MDA), maintaining ionic homeostasis (Na^+^/K^+^), and modulating stress-responsive gene expression. Together, these findings provide novel insights into the regulatory role of *GmRWP-RK1* in salt-stress responses and underscore its potential to improve stress resilience in soybean.

## 2. Results

### 2.1. Cloning of the GmRWP-RK1 and Stable Transformation in Arabidopsis

The *GmRWP-RK1* cDNA sequence was successfully obtained from the soybean W82 cultivar’s reference genome and subsequently cloned using gene-specific primers. A 981-bp fragment was amplified by PCR and inserted into the T1 vector, and the construct was confirmed by double restriction digestion with specific enzymes (Figure S1). The cDNA encodes an open reading frame of 326 amino acids, with a theoretical isoelectric point of 6.53 and a predicted molecular weight of 37.49 kDa. The vector (pCAMBIA3301) was digested with *BamHI* and *HindIII* (Figure S2), and the *GmRWP-RK1* gene was inserted into the digested sites. The plant over-expression vector pCAMBIA3301 was developed to enhance *GmRWP-RK1* expression in transgenic lines.

Homozygous T3 transgenic Arabidopsis seeds were grown in a growth chamber until reaching the flowering stage. Initial screening was conducted by spraying with a basta solution (20 µg mL^−1^) and using Bar rapid strips. Only robust plants were selected for RNA extraction and cDNA synthesis. The primer pair of *GmRWP RK1*-F/R (Table S1) was used to amplify the full coding region of *GmRWP-RK1* from the cDNA of transgenic plants. Six plants with a high copy number of *GmRWP-RK1* were identified by 1% agarose gel electrophoresis (Figure S3A). To confirm the presence of the pCAMBIA-*GmRWP-RK1* transgene, the herbicide resistance gene (Bar) was amplified (Figure S3B). Additionally, the presence of the nopaline synthase (NOS) terminator gene was verified (Figure S3C).

### 2.2. Over-Expression of GmRWP-RK1 in Arabidopsis Improves Salt Stress Tolerance

**Figure 1:**
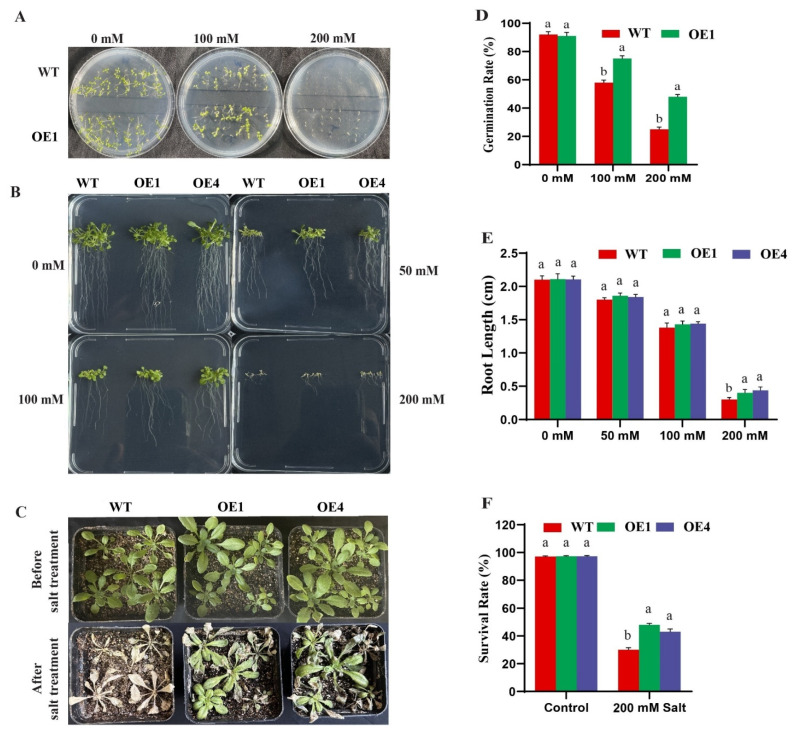
Salt tolerance phenotypes of wild-type (WT) and *GmRWP-RK1* overexpression (OE) Arabidopsis lines. (**A**) Germination of WT and OE1 seeds on MS medium supplemented with 0, 100, or 200 mM NaCl. (**B**) Primary root length of WT, OE1, and OE4 seedlings grown on half-strength MS medium containing 0, 50, 100, or 200 mM NaCl. (**C**) Representative phenotypes of WT and OE lines after 10 days of 200 mM NaCl treatment. (**D**–**F**) Quantification of germination, root length, and survival rate. Data are means ± SD of three independent biological replicates (n = 3). Different letters indicate significant differences determined by one-way ANOVA followed by Tukey’s multiple-comparison test (*p* < 0.05).

To assess how *GmRWP-RK1* transgenic plants respond to salt stress, two transgenic lines (OE1 and OE4) along with WT were selected for subsequent salt assays. A germination assay was performed on half-strength MS medium supplemented with 0, 100, and 200 mM NaCl (Figure 1A). Following 15 days of salt stress, WT seedlings exhibited noticeable growth inhibition at 100 mM NaCl, whereas the transgenic over-expression line (OE1) sustained relatively normal growth. Under severe salt stress (200 mM NaCl), the germination rate of WT was significantly reduced compared to OE1. Specifically, at 100 mM NaCl, OE1 achieved a germination rate of 75%, surpassing the 58% observed in WT. Even at 200 mM NaCl, OE1 maintained a 25% higher germination rate than WT (Figure 1D). Consistent with these findings, root length measurements on half-strength MS medium supplemented with varying concentrations of NaCl further demonstrated the greater performance of OE under salt stress (Figure 1B). Under control conditions (0 mM NaCl), root growth showed no significant difference between WT and OE lines. However, with increasing salt concentrations (50, 100, and 200 mM NaCl), root length decreased in both genotypes. Despite this overall reduction, OE plants consistently maintained longer roots than WT under salt stress. The disparity was particularly pronounced and statistically significant at the highest concentration (200 mM NaCl), suggesting that OE lines possess improved tolerance to salt-induced root growth inhibition (Figure 1E). To evaluate salt stress tolerance under soil conditions, 25-day-old Arabidopsis seedlings of WT and OE lines were subjected to 200 mM NaCl treatment for 10 days (Figure 1C). The transgenic lines OE1 and OE4 demonstrated enhanced tolerance, with survival rates of 48% and 43%, respectively, compared to 30% in WT plants (Figure 1F). These findings indicate that over-expression of *GmRWP-RK1* enhances salt stress tolerance in Arabidopsis, underscoring its potential role in mediating stress-responsive pathways.

### 2.3. Transgenic Lines Enhanced Physiological Responses and Reduced Stomatal Aperture Under Salt Stress

**Figure 2:**
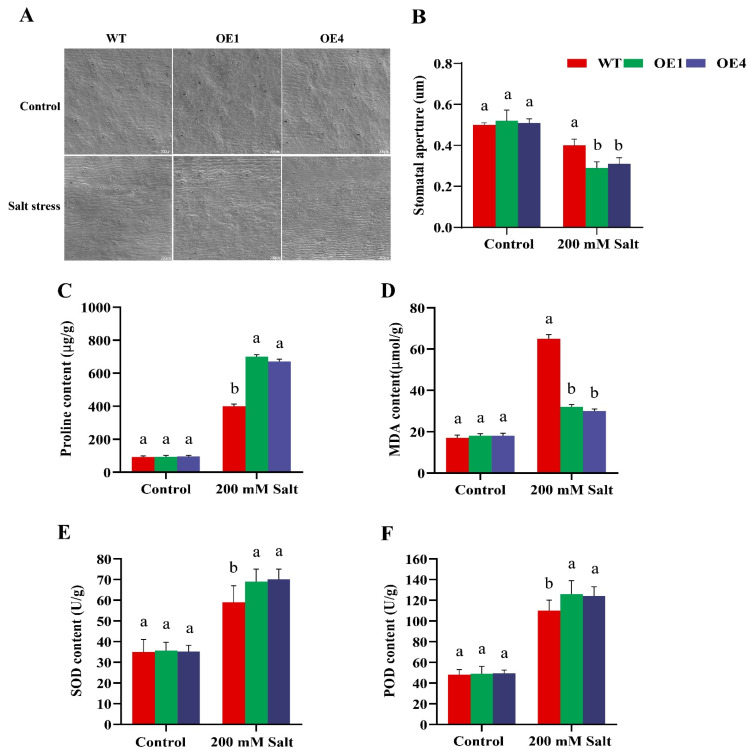
Physiological responses of WT and OE Arabidopsis lines under salt stress. Plants were evaluated for (**A**) stomatal morphology by SEM (scale bars 200 um), (**B**) stomatal aperture, (**C**) proline content, (**D**) MDA content, (**E**) SOD activity, and (**F**) POD activity under control and NaCl treatment conditions. Data are means ± SD of three independent biological replicates (n = 3). Different letters indicate significant differences determined by one-way ANOVA followed by Tukey’s test (*p* < 0.05).

To examine stomatal behavior and physiological responses, WT and OE lines were evaluated under control and salt stress conditions. After salt exposure, OE plants exhibited approximately 33.3% smaller stomatal apertures (width/length ratio) compared to WT. Representative microscopy images further demonstrated tighter stomatal closure in OE lines (Figure 2A,B). In addition, proline and MDA contents, along with the activities of SOD and POD, were measured to evaluate the physiological response under both conditions. Under normal conditions, there was no significant difference in proline content between WT and OE lines, which both showed relatively low levels. However, under salt stress, proline levels significantly increased in both groups. The WT group exhibited an average proline concentration of approximately 400 μg g^−1^, while the OE lines ranged from 730 to 750 μg g^−1^ (Figure 2C). MDA, a biomarker for oxidative stress-induced membrane damage, showed similar levels in both WT and OE plants under normal conditions. Under salt stress, however, MDA concentrations in WT plants doubled compared to those in the OE lines (Figure 2D). SOD and POD, key enzymes in the detoxification of superoxide radicals, showed similar activities in WT and OE plants under control conditions (Figure 2E,F). After salt treatment, SOD and POD activities were notably higher in the OE lines compared to WT, indicating an enhanced stress response.

### 2.4. GmRWP-RK1 Over-Expression Positively Regulated Photosynthesis, Biomass, and Ion Accumulation in Arabidopsis

**Figure 3:**
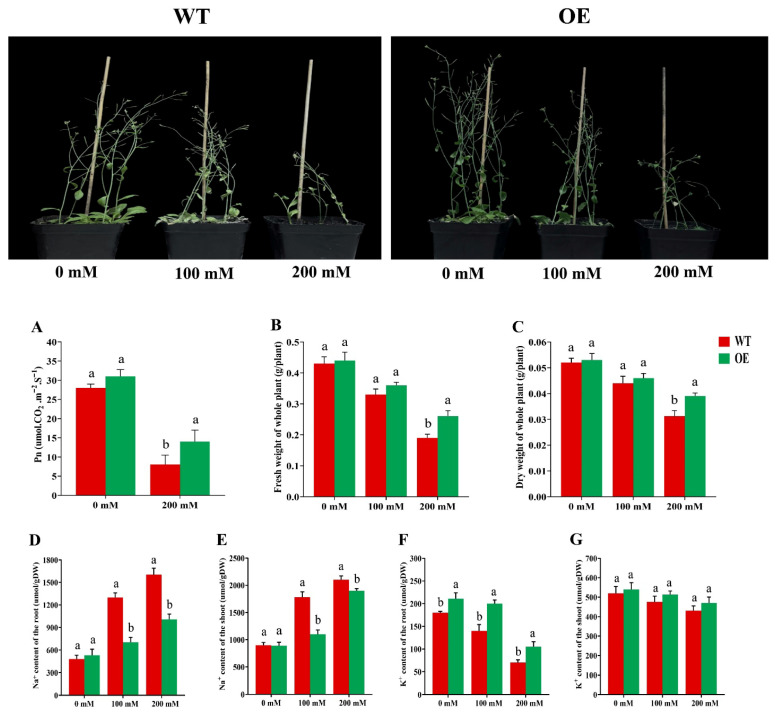
*GmRWP-RK1* overexpression improves photosynthetic performance, biomass accumulation, and ion homeostasis in Arabidopsis under salinity. Phenotypes of WT and OE plants under 0, 100, or 200 mM NaCl are shown together with quantitative measurements of (**A**) photosynthetic rate (Pn), (**B**) fresh weight, and (**C**) dry weight. Ion concentrations were measured by atomic absorption spectrophotometry: (**D**) Na^+^ in roots, (**E**) Na^+^ in shoots, (**F**) K^+^ in roots, and (**G**) K^+^ in shoots. Data are means ± SD of three independent biological replicates (n = 3). Different letters indicate significant differences determined by one-way ANOVA followed by Tukey’s test (*p* < 0.05).

Progeny from the T3 homozygous transgenic line (OE1), along with WT plants, were grown under salt and non-salt conditions as described above. Under salt stress, WT plants exhibited chlorosis, reduced leaf size, and overall growth inhibition, with these effects worsening as NaCl concentration increased. In contrast, OE plants were less affected and survived up to 200 mM NaCl (Figure 3). The results showed that NaCl treatment reduced photosynthetic rate (Pn), whereas Pn in transgenic plants was nearly double that in WT plants (Figure 3A). In both fresh and dry weight, NaCl treatment significantly reduced WT plant weight (Figure 3B,C). Collectively, the fresh and dry weights of OE plants were higher than those of WT plants. Furthermore, the ion contents (Na^+^ and K^+^) in both OE and WT plants were assessed before and after NaCl stress. Before exposure, Na^+^ content was similar in both WT and OE plants. NaCl treatment increased Na^+^ levels in both groups, but the Na^+^ content in the transgenic plants was significantly lower than in WT plants (Figure 3D,E). K^+^ contents in the shoots were comparable between WT and OE plants before salt treatment, while notable differences were observed in the roots. After NaCl exposure, K^+^ levels decreased in both WT and OE roots, with OE plants consistently retaining higher K^+^ levels (Figure 3F). NaCl treatment did not affect K^+^ content in the shoots of either WT or OE plants (Figure 3G).

### 2.5. AtRWP-RK5 (An Orthologue of GmRWP-RK1 in Arabidopsis) Significantly Induced the Expression of Its Interactor Genes Under Salinity Stress

**Figure 4:**
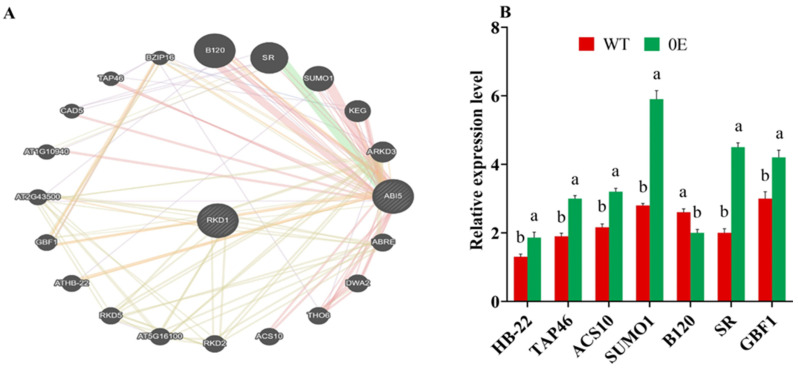
Predicted *AtRKD5* (*AtRWP-RK5*) regulatory network and expression of candidate interactor genes. (**A**) Protein–protein interaction (PPI) network of *GmRWP-RK1* and its putative Arabidopsis orthologue AtRKD5 inferred from the GeneMANIA platform. (**B**) RT-qPCR analysis of seven predicted network genes in WT and OE plants under control and NaCl stress. Data are means ± SD of three independent biological replicates (n = 3). Different letters indicate significant differences (*p* < 0.05).

*AtRWP-RK5*, also known as *AtRKD5* in Arabidopsis, is the ortholog of *GmRWP-RK1*. To determine the effect of *GmRWP-RK1* overexpression on their interactor genes, we first investigated the regulatory network and key interactor partner genes of AtRKD5 (renamed RKD1) using the GeneMANIA database (Figure 4A). To experimentally test this hypothesis, we conducted an expression analysis of seven interactor genes identified through GeneMANIA predictions using *GmRWP-RK1* over-expression transgenic lines. The expression results showed that *AtHB22*, a key regulator of NEPG and PEG expression under stress, was significantly up-regulated in transgenic plants compared to WT under salt stress (Figure 4B). Similarly, *AtTAP46*, a conserved component of the TOR signaling pathway, showed increased expression in transgenic plants under salt stress. Concurrently, *AtACS10*, which encodes aminocyclopropane-1-carboxylate synthase 10, was also up-regulated in transgenic plants when compared to WT. Noticeably, the expression of *AtSUMO1* (SMALL UBIQUITIN-LIKE MODIFIER 1), *AtSR*, and *AtGBF1* (G box-binding factor 1 (*bZIP*) showed the most prominent upregulation in transgenic plants compared to the WT when exposed to salt stress. In contrast, the interactor gene *B120*, a protein kinase gene associated with pollen recognition, was downregulated in transgenic plants compared to WT. These findings indicate that *GmRWP-RK1* overexpression enhances the transcriptional activation of multiple *AtRKD5*/*AtRWP-RK5* interactor genes under salinity stress, indicating an association with stress-responsive signaling processes.

### 2.6. Genetic Evidence Supporting the Role of GmRWP-RK1 in Salt Stress Tolerance Using the Hairy Roots System in Soybean

**Figure 5:**
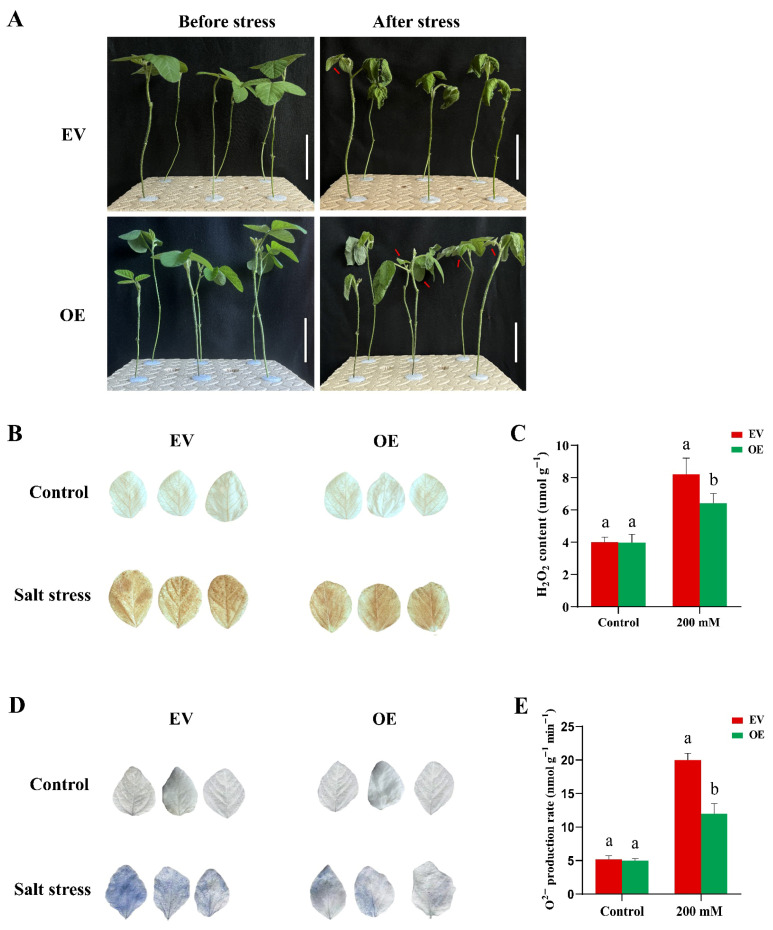
Salt tolerance and ROS accumulation in chimeric soybean plants carrying empty vector (EV) or *GmRWP-RK1* overexpression (OE) hairy roots. (**A**) Representative EV and OE plants before and after 200 mM NaCl treatment; red arrows indicate leaf damage (scale bar 5 cm). (**B**) DAB staining for H_2_O_2_ and (**D**) NBT staining for O^2−^ in leaves before and after salt stress. (**C**,**E**) Quantification of staining intensities. Data are means ± SD of three independent biological replicates (n = 3). Different letters indicate significant differences determined by one-way ANOVA followed by Tukey’s test (*p* < 0.05).

To functionally validate the role of *GmRWP-RK1* in salt stress tolerance, we generated chimeric soybean plants overexpressing *GmRWP-RK1* in transgenic hairy roots (OE) alongside empty vector controls (EV). Once the transgenic roots reached 8–10 cm in length, both EV and OE plants were transferred from vermiculite to Hoagland nutrient solution for a 5-day acclimation period (Figure 5A). Following acclimation, plants were subjected to 200 mM NaCl for 10 days to assess their phenotypic and physiological response to salinity stress. Upon salt exposure, all plants demonstrated typical stress-associated symptoms, including chlorosis, leaf wilting, reduced leaf area, and premature leaf drooping (Figure 5A). However, EV plants showed more severe leaf burn than OE plants, indicating that over-expression of *GmRWP-RK1* in the roots enhances salt tolerance, likely by reducing Na^+^ uptake, which in turn supports the growth of the non-transgenic aerial portions of the OE chimeric soybean plants more effectively than in the EV plants.

Moreover, chimeric transgenic plants (OE) exhibited reduced accumulation of the two primary ROS contents (H_2_O_2_ and O_2_^−^) than the EV plants following salt stress (Figure 5B–E). After 10 days of exposure to 200 mM NaCl, both OE and EV plant leaves were stained with DAB and NBT to visualize in situ accumulation of H_2_O_2_ and O_2_^−^. Under normal conditions, there were no significant differences between EV and OE leaves. However, after salt treatment, a visible increase in ROS levels was observed in both OE and EV leaves. Notably, the EV leaves accumulated significantly higher levels of H_2_O_2_ and O_2_^−^, as evidenced by the darker brown (Figure 5B) and blue (Figure 5D) staining. In contrast, the OE lines exhibited lower levels of H_2_O_2_ and O_2_^−^ compared to the EV plants (Figure 5C,E). This suggests that under stress conditions, OE transgenic roots help reduce ROS accumulation in the non-transgenic aerial parts, thereby conferring improved stress tolerance compared to EV plants.

### 2.7. GmRWP-RK1 Overexpressed Transgenic Hairy Roots in Soybean Improved Antioxidant System and Ionic Balance Under Salt Stress

**Figure 6:**
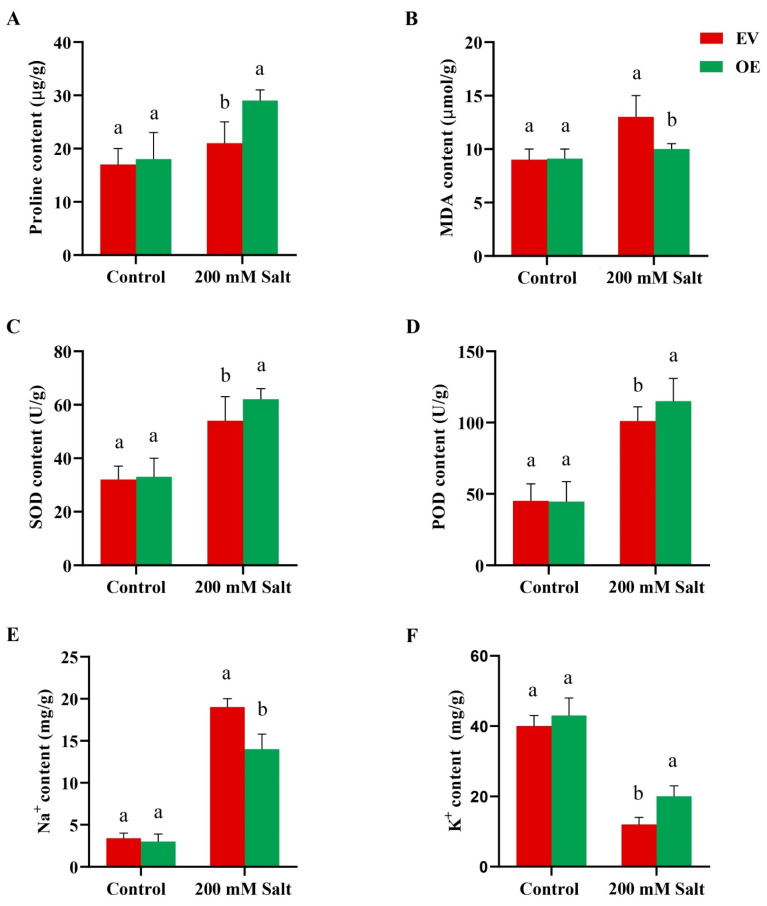
Antioxidant activities and ion contents in EV and OE soybean hairy roots before and after salt stress. (**A**) Proline, (**B**) MDA, (**C**) SOD, (**D**) POD, (**E**) Na^+^, and (**F**) K^+^ contents. Data are means ± SD of three independent biological replicates (n = 3). Different letters indicate significant differences determined by one-way ANOVA followed by Tukey’s test (*p* < 0.05).

Proline and MDA levels, as well as SOD and POD activities, were measured to assess the physiological effects of *GmRWP-RK1* over-expression in chimeric soybean plants. Under control conditions (Hoagland solution), proline levels were similar in both EV and OE roots. However, under salt stress, proline content significantly increased in OE hairy roots (Figure 6A). MDA levels were slightly higher in OE roots than in EV roots under control conditions, but after salt treatment, MDA levels in EV roots spiked considerably compared to OE roots (Figure 6B). SOD activity was significantly elevated in OE hairy roots relative to EV roots under salt stress conditions (Figure 6C). Conversely, POD activity was greater in EV roots than in OE roots under salt stress (Figure 6D). In addition, the ion contents in both OE and EV hairy roots were measured before and after salt exposure, focusing on Na^+^ and K^+^ due to their critical roles in the salt stress response. Before salt treatment, Na^+^ content was similar in both EV and OE roots. As expected, salt treatment increased Na^+^ levels in both root types; however, Na^+^ content was significantly lower in OE roots compared to EV roots (Figure 6E). Before salt stress, K^+^ content was slightly higher in OE roots. After salt treatment, K^+^ content decreased in both EV and OE roots, but OE roots consistently maintained higher K^+^ levels than EV roots (Figure 6F). Together, these findings suggest that over-expression of *GmRWP-RK1* enhances salt tolerance in chimeric plants by modulating the antioxidant system and Na^+^/K^+^ ratio.

### 2.8. Phenotypic and Anatomical Evaluation of EV and OE Hairy Roots Under Salt Stress

**Figure 7:**
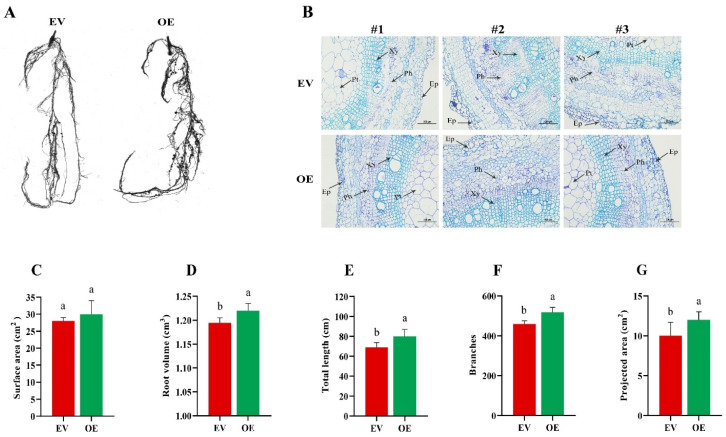
Root architectural traits and anatomical features of EV and OE soybean hairy roots under salt stress. (**A**) Representative scanned images of hairy roots. (**B**) Transverse sections from different root regions (#1–3); Ep, epidermis; Ph, phloem; Xy, xylem; Pt, pith (scale bars 100 um). (**C**) Surface area, (**D**) root volume, (**E**) total length, (**F**) number of branches, and (**G**) projected area of EV and OE roots. Data are means ± SD of three independent biological replicates (n = 3). Different letters indicate significant differences determined by one-way ANOVA followed by Tukey’s test (*p* < 0.05).

To assess the impact of salt stress on soybean root microstructure, anatomical analyses were performed after 10 days of salt treatment. The root structure of EV plants showed significant alterations (Figure 7B). The epidermal layer exhibited inward and outward depressions, with some cells bursting due to dehydration. The central cells were also adversely affected. In contrast, the root structure of the OE strain exhibited limited disruption, with cells organized more orderly and no significant changes in the central cells (Figure 7B). While the root epidermis of both EV and OE roots did not show notable differences after salt treatment, the xylem in the EV roots exhibited a substantial reduction, with some cells bursting, and the phloem cells became slimmer. In contrast, the OE strains exhibited notably thicker xylem and phloem than the control plants. To conclude, the improved salt tolerance in the OE strains appears to be associated with a reduction in the thickness of the root epidermis and cortex, alongside an enhancement in the thickness of the xylem and phloem cells. The roots were analyzed using a root scanner machine (Figure 7A). Significant differences were observed between OE and EV roots in most measured parameters, including root volume (Figure 7D), total root length (Figure 7E), number of branches and projected area (Figure 7F), except for root surface area (Figure 7C). Overall, these anatomical and morphological improvements suggest that *GmRWP-RK1* overexpression enhances root structural integrity and growth performance under salt stress.

### 2.9. GmRWP-RK1 Over-Expression Regulated Downstream Genes in Soybean Hairy Roots Under Salt Stress

**Figure 8:**
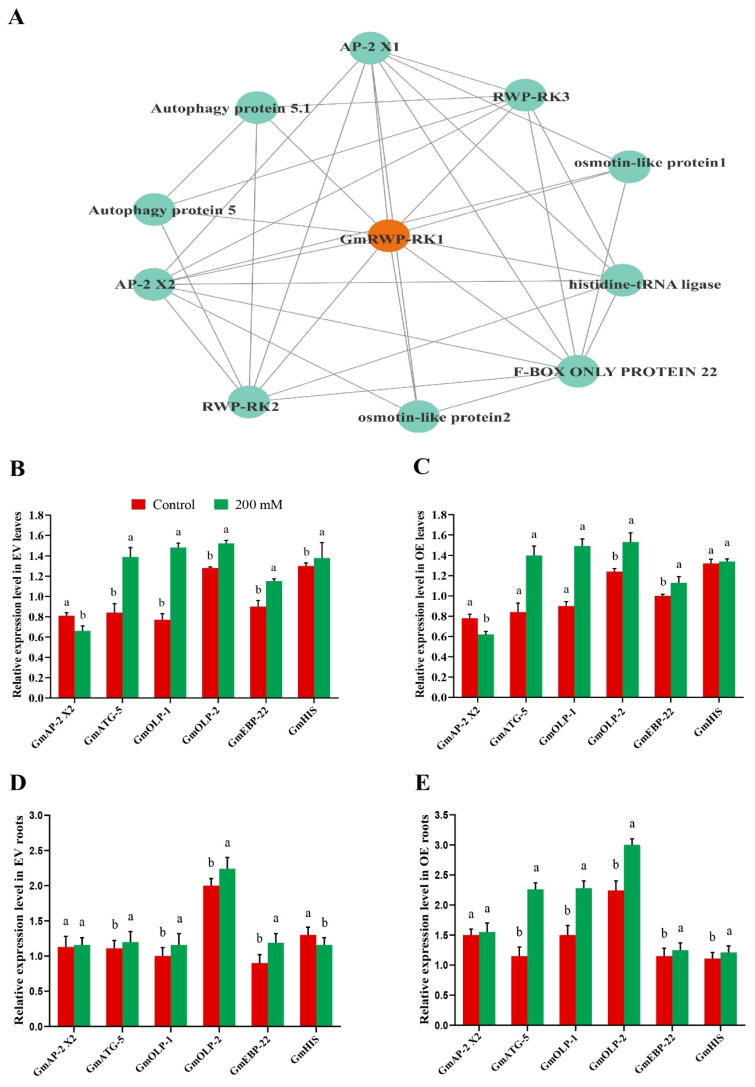
Expression analysis of the *GmRWP-RK1*-centered regulatory network in chimeric soybean plants under salt stress. (**A**) Predicted PPI network of *GmRWP-RK1*. RT-qPCR expression profiling of selected interacting genes in (**B**) EV leaves, (**C**) OE leaves, (**D**) EV roots, and (**E**) OE roots. Data are means ± SD of three independent biological replicates (n = 3). Different letters indicate significant differences (*p* < 0.05).

To explore *GmRWP-RK1*-induced expression of other interactor genes in soybean, we conducted expression analysis of 10 genes identified using the STRING database (Figure 8A). Of these ten genes, we select six interactor genes based on their strong interaction with *GmRWP-RK1*. The results showed that these genes exhibited distinct expression levels in chimeric soybean roots under control and salt stress conditions (Figure 8D). For instance, *GmAp2* (Ap2 complex subunit alpha-1 isoform X2) showed slight upregulation under salt stress in EV roots, while its expression remained unchanged in OE roots (Figure 8E). Under salt stress, plants can activate selective autophagy pathways to mitigate stress. In this context, *GmATG5* (autophagy protein 5) was up-regulated in EV roots during salt stress. Interestingly, the expression of *GmATG5* in OE roots was twice as high as in EV roots. Osmotin-like proteins (OLPs), which play a potential role in osmotic regulation and stress response, showed substantial upregulation in response to salt stress. Both *GmOLPs-1* and *GmOLPs-2* showed elevated expression in both EV and OE roots, *GmOLPs-2* exhibited a two-fold higher expression in *GmRWP-RK1*-OE roots compared to EV roots under salt stress. *GmEBP-22* (E-box binding protein 22), a member of the ERF family known to regulate stress-responsive genes, was significantly up-regulated in both EV and OE roots under salt stress. Histidine-tRNA ligase, which maintains cellular osmotic balance by attaching histidine to tRNA during translation, displayed contrasting regulation. In EV roots, the expression of histidine-tRNA ligase was downregulated, whereas it was significantly up-regulated in *GmRWP-RK1*-OE roots under salt stress. In contrast to the roots, the expression of these genes in the EV leaves are similar to the pattern observed in the OE leaves of soybean under salt stress (Figure 8B,C). Together, these results demonstrate that *GmRWP-RK1* overexpression positively rewires the downstream stress-responsive regulatory network in soybean hairy roots, enhancing the induction of key osmotic, autophagy-related, and defense-related genes under salinity. Collectively, these coordinated transcriptional changes suggest *GmRWP-RK1* may act as an important regulator of adaptive responses to salt stress in soybean.

## 3. Discussion

Salt stress causes osmotic and ionic imbalance that promotes the accumulation of reactive oxygen species (ROS), damaging proteins, lipids, and DNA, and ultimately restricting plant growth [22]. Because ROS rise under salinity, stress tolerance depends on maintaining a balance between ROS production and detoxification [23]. Multiple stress-responsive gene families contribute to this control [24,25,26]. For example, over-expression of *OsHsfC1b* improves salt tolerance in rice [27]. Members of the *RWP-RK* family, which has been linked to phenylpropanoid biosynthesis, can also support stress tolerance by enhancing free-radical scavenging capacity [28]. In Arabidopsis, *AtRKD5* (*AtRWP-RK5*), a close ortholog of *GmRWP-RK1*, has been associated with abiotic stress-responsive regulation and nitrogen-related signaling [14,16]. Nitrogen signaling can intersect with salt stress via nitric oxide (NO), which accumulates under stress and may be generated through nitrate reductase activity [29,30]. In wheat, NO treatment promotes stomatal closure, improves osmotic tolerance [31] and reduces water loss while increasing ABA levels [32].

Considering previous literature and our prior screening of soybean *RWP-RK* candidates, we selected *GmRWP-RK1* for functional characterization. We cloned *GmRWP-RK1*, constructed an over-expression vector (pCAMBIA-*GmRWP-RK1*), and generated transgenic Arabidopsis lines and soybean hairy-root chimeras. *GmRWP-RK1* over-expression enhanced salt tolerance, with transgenic Arabidopsis showing improved germination and survival under salinity compared with wild-type plants (Figure 1). Consistent with improved oxidative stress management, histochemical staining and ROS measurements showed that *GmRWP-RK1* over-expression (OE) chimeric plants scavenged ROS more efficiently than EV (Figure 5B–E). OE soybean hairy roots also displayed that *GmRWP-RK1* OE produces a larger, more highly branched root system than the EV (Figure 7A). The representative root images and root cross-sections indicate stronger root development in OE, and the quantitative traits confirm significant increases in root surface area, root volume, total root length, branch number, and projected area (Figure 7A), suggesting that *GmRWP-RK1* may influence root physiological status under stress. Overall, these results indicate that *GmRWP-RK1* contributes to salinity resilience, at least in part, by supporting root growth and performance under adverse conditions.

Enhanced antioxidant capacity is a common feature of stress-tolerant genotypes, and over-expression of transcription factors such as *SlbHLH22* and *PavbHLH28* can increase the activities of antioxidant enzymes (SOD, CAT and POD), thereby improving stress resilience [33]. Similarly, *OsHsfA7*-overexpressing rice seedlings show reduced stress injury, as evidenced by lower relative electrolyte conductivity (REC) and malondialdehyde (MDA), reflecting decreased membrane damage and lipid peroxidation [34,35]. In our study, transgenic Arabidopsis accumulated higher proline and showed lower MDA levels under salt stress, while SOD and POD activities were significantly higher than in WT plants (Figure 2), supporting a role for *GmRWP-RK1* in strengthening antioxidant defenses. Ion homeostasis is also central to salt tolerance because Na^+^ disrupts K^+^ influx and cellular functions [36]. *GmRWP-RK1* lines maintained more stable Na^+^ and K^+^ levels and a lower cytosolic Na^+^/K^+^ ratio than controls (Figure 3), consistent with classical models of salt tolerance [37,38]. In soybean hairy roots, POD activity differed only marginally between EV and OE lines under control conditions (Figure 6). Following salt stress, POD activity was significantly higher in OE lines than in EV controls, consistent with enhanced activation of antioxidant defenses. Likewise, SOD activity was significantly increased in OE lines, supporting improved ROS-scavenging capacity under salinity (Figure 6). These enzymatic changes were accompanied by higher proline accumulation, lower MDA levels, and a reduced Na^+^/K^+^ imbalance in OE roots relative to EV controls (Figure 6), collectively supporting a role for *GmRWP-RK1* in promoting redox homeostasis, ionic regulation, and stress protection [39].

To explore the molecular mechanisms underlying these phenotypes, we examined gene expression within the *GmRWP-RK1* regulatory network. In Arabidopsis, *GmRWP-RK1* over-expression modulated stress-responsive transcripts including *AtHB22*, *AtTAP46* and *AtACS10* (Figure 4), which function in stress signaling, hormone-related responses and transcriptional regulation [40,41,42]. In soybean, *GmRWP-RK1* increased salt-induced expression of *GmATG-5*, *GmOLP-1* and *GmOLP-2* (Figure 8), genes implicated in protective processes such as autophagy and cell wall-associated responses [43,44]. *GmRWP-RK1* therefore appears to act as an upstream regulator that coordinates transcriptional responses supporting redox balance and ion homeostasis under salinity; however, proposed links to specific hormonal or nodulation signaling remain putative because we did not measure hormone levels, canonical marker genes or signaling outputs. The predicted miRNA network (Figure S5, Table S4) further suggests a potential post-transcriptional layer that could refine stress-induced transcript dynamics and downstream redox/ion regulation, and representative interactions should be validated in future work. Our findings are consistent with emerging evidence that *RWP-RK* transcription factors contribute to abiotic stress tolerance beyond their established roles in nitrogen signaling: *NLP*-mediated nitrate signaling has been connected to salt tolerance in rice via the *OsNLP4-OsMADS27* module [20], and *RWP-RK* genes in pearl millet and elephant grass have been associated with heat adaptability and antioxidant responses [14]. We acknowledge that 200 mM NaCl is an extreme screening condition; thus, our conclusions should not be overgeneralized to field salinity and require validation under moderate, agronomically relevant conditions. We used *Arabidopsis* as a rapid, genetically tractable heterologous system for initial functional validation of *GmRWP-RK1*, given that generating stable transgenic soybean lines is time-consuming and technically challenging. To strengthen soybean relevance, we complemented the Arabidopsis analyses with soybean hairy-root assays to evaluate root physiological traits in soybean tissues. However, because the soybean hairy-root system provides rapid, root-focused assays but does not fully capture whole-plant or multi-generation effects, future work should validate *GmRWP-RK1* function in stable soybean transgenic or genome-edited lines and assess performance under greenhouse and field salinity conditions.

## 4. Materials and Methods

### 4.1. RNA Extraction and Cloning of the GmRWP-RK1 Gene

To obtain the complete cDNA sequence of *GmRWP-RK1,* we employed the Williams 82 cultivar genome sequence. Samples were collected and immediately flash-frozen in liquid nitrogen. Total RNA was extracted using RNA Iso-Plus (Takara, Beijing, China), and cDNA was synthesized through reverse transcription PCR using Super Script IV reverse transcriptase (Thermo Scientific, Beijing, China). Gene-specific primers were used for PCR amplification. The forward primer was designed with a *BamHI* recognition site. It included a start codon to initiate translation, while the reverse primer contained a *HindIII* restriction site immediately after the stop codon. Primers were used, i.e., RWP1-F: 5′GGGGGAGGACCTCTAGATGTGCTGGATACTGTTGAATTG3′ and RWP1-R: 5′GGAGGACCTCTAGATGTGCTGGATACTGTTGAATTG3′. PCR amplification of the *GmRWP-RK1* was carried out with Pfu DNA polymerase (Takara, Beijing, China). The amplified product was then cloned into the T-vector (pEASY-T1) (Takara, Beijing, China), and the recombinant construct was introduced into *E. coli* (TransT1) competent cells by heat shock transformation method and then sent for sequencing.

### 4.2. Construction of Over-Expression Vector and Detection of Transgenic Lines

The entire coding sequence of *GmRWP-RK1* was amplified using primers specific to the *GmRWP-RK1* gene, as described previously. The amplified coding region was then inserted into the pCAMBIA3301 vector, which had been previously digested with BamHI and HindIII and contained the Bar gene under the control of the 35S (CaMV) promoter [45]. The *GmRWP-RK1* gene and the plant over-expression vector were ligated using T4 ligase. The recombinant construct was transformed into DH5α *E. coli* competent cells via heat shock, and positive colonies were identified by colony PCR and confirmed by Sanger sequencing [46]. The recombinant pCAMBIA-*RWPRK1* vector was subsequently transformed into *Agrobacterium tumefaciens* (EHA105 strain). Wild-type *Arabidopsis thaliana* was genetically transformed using a modified floral-dip technique [47]. T1 seeds were selected with BASTA herbicide, and transgenic plants were propagated through the T2 and T3 generations. Transgenic plants were screened by PCR amplification of the *GmRWP-RK1* gene, the herbicide resistance gene (Bar), and the TNOS gene using GoTaq DNA polymerase with specific primer sets.

### 4.3. Salt Treatment of Transgenic Arabidopsis Plants

For salt treatment, young WT and T3 transgenic Arabidopsis seedlings (OE1, OE4) grown in plastic pots were treated with 200 mM NaCl. The survival rate was inspected after 10 days of salt treatment. Furthermore, to assess the impact of salt stress on seed germination, seeds from WT and the OE1 line were used. OE1 was selected based on its stable transgene expression and a representative salt-tolerant phenotype consistent with the other OE lines. Seeds were surface-sterilized by immersion in 75% ethanol for 1 min, followed by 1% sodium hypochlorite for 5–10 min, and then rinsed 3–6 times with sterile distilled water, as previously [48]. The sterilized seeds were cultured on half MS medium supplemented with 100 and 200 mM NaCl and a control. The count of germinated seeds was conducted 15 days post-sowing. For root length analysis, sterilized seeds were germinated on a half-strength MS medium under aseptic conditions. After 7 days of germination, uniformly sized seedlings were directly transferred to vertical square plates containing MS agar medium supplemented with 0 (control), 50, 100, and 200 mM NaCl to impose abrupt salt stress (seedlings were moved directly onto medium already containing the final NaCl concentration). Plates were placed vertically, and the root growth was observed and documented after one month [48].

### 4.4. Antioxidant Enzymatic Activity and MDA Content

The proline content in the WT and OE lines was determined following the method of [49]. MDA levels, an indicator of lipid peroxidation, were quantified through the thiobarbituric acid (TBA) assay, as outlined by [50]. SOD activity was measured using the method developed by [28], which relies on the enzyme’s ability to inhibit the photochemical reduction of nitro-blue tetrazolium (NBT). POD activity was measured using the method described by [51]. The reaction mixture contained 50 mL of 100 mM potassium phosphate buffer (pH 6.0), 30 μL of 0.3 mM guaiacol, and 20 μL of 30% H_2_O_2_. To initiate the reaction, 3 mL of the mixture and 15 μL of enzyme extract were added to a colorimetric cuvette. Absorbance values were measured at 240 nm every 30 s for 5 readings. POD activity was determined by calculating the rate of absorbance change per minute.

### 4.5. Quantification of Total Na^+^ and K^+^ Ions in Transgenic Plants Under Salt Stress

Transgenic Arabidopsis and soybean plants were assessed for measuring their Na^+^ and K^+^ ratio. Arabidopsis seeds were planted in plastic pots that contained a 2:1 (*v*/*v*) mix of compost and vermiculite. Approximately 40-day-old Arabidopsis plants were exposed to salt stress, and each pot received 60 mL of Hoagland solution every other day for 10 days. The NaCl-supplemented concentrations gradually increased by 50 mM every 2 days for each group until reaching their respective maximum levels as indicated. Moreover, for soybean plants comprising OE and EV, hairy roots were assessed for their salt tolerance and were subjected to 200 mM salt stress in Hoagland solution for 10 days. After salt stress, samples were washed and placed in an oven at 60–70 °C for 72 h. The samples were digested in nitric acid (HNO_3_), heated until dissolved, and then diluted to 25 mL with deionized water before filtering. Na^+^ and K^+^ concentrations were determined using an atomic absorption spectrophotometer (Z-8000, Hitachi, Tokyo, Japan) by following the method described by [52], calibrated with known standards, and normalized to dry weight for comparison between treatments. The recorded data were presented as the grand mean standard error across three replicates (n = 3).

### 4.6. Assessment of Photosynthetic Rate and Fresh and Dry Weight of Transgenic Plants Under Salt Stress

After NaCl treatment, plant growth was evaluated by measuring photosynthetic rate and fresh and dry weight in transgenic plants overexpressed with *GmRWP-RK1*. Fresh weight measurements were taken immediately following harvesting, while dry weight was assessed after drying the plants at 62 °C for 48 h [53].

### 4.7. Expression Profiling of RWP-RK1 Interactor Genes in Arabidopsis Under Salt Stress

To test the expression induction of *GmRWP-RK1* interactor genes in Arabidopsis under salt stress, we first conducted a computational approach to predict the regulatory network for *AtRKD1* (the Arabidopsis orthologue of *GmRWP-RK1*), using the GeneMANIA database (http://genemania.org). We selected seven interactor genes and analyzed their expression to assess the impact of *GmRWP-RK1* overexpression in WT and OE plants under salt stress. Briefly, RNA was extracted using RNAiso Plus (Takara), followed by cDNA synthesis with the Takara RT kit. Then, RT-qPCR was performed in triplicate for each sample, with *Actin 11* (AtACT11; locus AT3G12110) used as an internal control [48]. Relative expression levels were calculated using the 2^−ΔΔCt^ method [54]. Table S2 lists the primers for RT-qPCR of seven genes, designed using Primer BLAST.

### 4.8. Soybean Hairy Root Transformation with GmRWP-RK1

For hairy root development, viable seeds were collected and sterilized with a Cl_2_ solution (25 mL hypochlorite and 5 mL HCl). The seeds were then placed in a fume hood for 16 h. Furthermore, the seeds were planted in pots filled with sterilized vermiculite and maintained in a growth chamber at 28 °C under a 16 h photoperiodism with regular watering. Simultaneously, the K599 strain from the glycerol stock was streaked onto YEP Petri plates containing kanamycin and incubated at 28 °C for 48 h. After that, a single clone was taken, and the mixture was incubated in a shaker at 250 rpm for 14 h at 28 °C. A 150 μL aliquot of the culture inoculum was spread onto YEP petri plates supplemented with kanamycin, incubated at 28 °C for 46 h, and subsequently used for transformation. When the soybean seedlings were ready for sprouting, cotyledons unfolded, but unifoliate leaves remained undeveloped, five-day-old seedlings were infected with the K599 strain carrying the *GmRWP-RK1* over-expression construct. The *Agrobacterium rhizogenic* culture was collected, sub-cultured, and introduced into the hypocotyl near the cotyledonary node, as described by [55]. K599 containing the empty vector (EV) was used to regenerate hairy roots for control plants. After transformation, seedlings were covered with a polytene tray to maintain humidity. Hairy roots appeared at infection sites within 2–3 weeks. Two weeks later, the roots were covered with sterilized vermiculite and kept moist. Once the hairy roots were fully grown, the detection of the EV and OE constructs was confirmed by extracting DNA from both root types and performing PCR with Bar gene primers. Additionally, the roots were scanned using a root scanner, and the data were processed using appropriate software.

### 4.9. Salt Assay of Transgenic Hairy Root Plants in Hoagland Solution

Once the hairy roots reached 8–10 cm in length, the soybean plants were carefully uprooted, and their primary roots were displayed. The resulting chimeric plants, featuring transgenic roots and non-transgenic shoots, were transplanted into fresh vermiculite and kept consistently moist through regular watering. Notably, the shoots of each plant were pruned after the emergence of the second pair of trifoliate leaves. New leaves after pruning were compared between chimeric EV and OE plants, which were then transferred to Hoagland solution to assess transgenic root performance under salt stress. After a 4-day acclimatization period, the plants were treated with 200 mM NaCl.

### 4.10. Histochemical and Anatomical Assay of Chimeric Soybean Under Salt Stress

The presence of O_2_^−^ and H_2_O_2_ under normal and salt stress conditions was detected by histochemical staining using nitro blue tetrazolium (NBT) and 3,3′-diaminobenzidine (DAB) as described by [56] and [57]. For O_2_^−^ detection, leaves from both EV and OE plants were submerged in a 1 mg mL^−1^ NBT solution (pH 7.8) at room temperature in the dark for 3–4 h. For H_2_O_2_ detection, leaves were immersed in a fresh 1 mg mL^−1^ DAB solution (pH 3.8) for 6–8 h. The stained leaves were then washed with sterile water, immersed in 95% ethanol, and decolorized in a 75 °C water bath for 15–20 min. To quantify O_2_^−^ and H_2_O_2_ levels, the stained leaves were homogenized in potassium phosphate buffer, incubated, and centrifuged at 12,000 rpm for 10 min. The obtained supernatant was examined using a spectrophotometer, with absorbance measured at 530 nm for O_2_^−^ and 485 nm for H_2_O_2_, and concentrations were determined using standard curves. These absorbance values corresponded to levels of superoxide radicals and hydrogen peroxide in leaf tissues.

The root anatomy of soybean plants was examined using a paraffin embedding technique, with slight modifications based on the procedure described by [58]. After 10 days of 200 mM salt stress, the chimeric soybean roots (EV, OE) were carefully removed and thoroughly rinsed with water. A cross-sectional root segment was excised from the central region of the primary root of three different sections. The root samples were promptly fixed in formalin-acetic acid (FAA) for at least 50 hrs, and 16 µM thin sections were cut using a microtome (Leica RM2245, Wetzlar, Germany). Toluidine blue-stained paraffin sections were examined and photographed using a fluorescence microscope (Nikon ECLIPSE, Tokyo, Japan).

### 4.11. Expression Analysis of the Regulatory Network GmRWP-RK1 in Chimeric Soybean Plants

The STRING database was used to predict potential interactors of *GmRWP-RK1* in soybean. Primers for RT-qPCR targeting six genes were designed using Primer-BLAST (Table S3). RNA from EV and OE samples was isolated using RNAiso Plus (Takara) and was assessed to determine the impact of *GmRWP-RK1* over-expression on its interacting proteins. After ensuring high RNA quality, cDNA was synthesized using the Takara RT kit. RT-qPCR was performed in triplicate, using *Actin11* (GmACT11; gene model Glyma.18G290800) as an internal control. Gene expression levels were calculated using the 2^−ΔΔCt^ method [54].

### 4.12. Statistical Analysis

The experiments were performed with three independent biological replicates (n = 3). Data are presented as mean ± SD. For comparisons among multiple groups, one-way ANOVA followed by Tukey’s multiple-comparison test was applied. Differences were considered statistically significant at *p* < 0.05 [59].

## 5. Conclusions

In this study, the phenotypic, physiological, and molecular analyses of both transgenic *Arabidopsis* and chimeric soybean plants revealed that *GmRWP-RK1* plays an important role in salt stress responses. When roots were subjected to salt stress, over-expression of *GmRWP-RK1* led to the accumulation of oxidative stress markers, which helped scavenge excess ROS and protect cell membrane integrity. Similarly, it might help regulate Na^+^ and K^+^ transport to maintain ionic homeostasis and osmotic balance. Additionally, the expression levels of stress-responsive genes in *GmRWP-RK1*-overexpression *Arabidopsis* and chimeric soybean plants were notably higher than those in non-transgenic plants under salt conditions. These findings suggest that *GmRWP-RK1* may function as an enhancer in salt tolerance, improving the plant’s ability to cope with high salinity.

## Data Availability

The original contributions presented in this study are included in the article/Supplementary Materials. Further inquiries can be directed to the corresponding authors.

## Supplementary Materials

The following supporting information can be downloaded at: https://www.mdpi.com/article/10.3390/plants15060912/s1, Figure S1: Molecular cloning and confirmation of the recombinant plasmid. Figure S2: Construction of plant overexpression vector. Schematic illustration of the expression vector. Figure S3: Molecular detection of transgenic plants. Figure S4: Different stages of the soybean hairy root transformation. Figure S5: Detection of transgenic hairy roots of soybean. Figure S6: miRNAs prediction network of *GmRWP-RK* transcripts. Figure S7: Schematic diagram of the molecular components of the cloning vector. Table S1: List of primers used for PCR implication in transgenic plants. Table S2: Primers for the qRT-PCR of *GmRWP-RK1* orthologue’s regulatory network in *Arabidopsis*. Table S3: Primers for the qRT-PCR of *GmRWP-RK1* regulatory network in soybean. Table S4: miRNA potential targets in *GmRWP-RK* transcripts.
