# Supplementary material for: GmRWP-RK1 Enhances Salt Tolerance by Modulating Antioxidant Defense, Ion Homeostasis and Stress-Responsive Pathways in Soybean
Source: Plants (Basel). 2026 Mar 16;15(6):912. doi: 10.3390/plants15060912 (PMC13029631; doi:10.3390/plants15060912)
Supplement: Supplementary file 1 [file plants-15-00912-s001.zip › plants-4109508-supplementary.pdf]

Supplementary Data

GmRWP-RK1 Enhances Salt Tolerance by Modulating Antioxidant Defense, Ions Homeostasis and Stress-Responsive Pathways in Soybean

Liu Lu [1†], Bai Qianyue[1†], Xu Min[1], Zhang Qi[1], Gai yuhong[1], Naveed Ahmad[2], Piwu Wang[1], Zhang Zhuo[1*], Nooral Amin[1,2*], and Wei JianJiang[1,2*]

1 Plant Biotechnology Centre, College of Agronomy, Jilin Agricultural University, Changchun 130118, China

2 Institute for Safflower Industry Research of Shihezi University/Pharmacy College of Shihezi University/Key Laboratory of Xinjiang Phytomedicine Resource and Utilization, Ministry of Education, Shihezi, 832003, China

>GmRWP-RK1 (Glyma.01G159200) Gene CDS sequence: (981 bp)

ATGGAGTCCACTTTGCTCACCAATCTACTAGTCTTCAACAACACCCTCCAACCAGAGTTAATGAGGAG
TGTGCATGTGTACCGCCGAGGGGATGGAGAGAAAAGGGAAGTTGAGAGAGAGTTTGTGTTTTCAGAG
AGCGGCTCCTATGGGGAAATGCAGGCTACCCCAATTCCTGGATTGGTAAAATCTCGTGTTTCTGAAGT
GTGTGAAGGGCACCGAAATGGAGTGTGGCTTTGTGTCTTTGCGTTCCATGCCGATCACACTCCTCAATT
TTGCCGCATTCCACCGGTTTTGTTGGTCACAAGAAACCCAAAGCTTAAGATGATTCCAAACTTGCTTGA
TGATCTCCATGTGATATACAAGTTGGACCGGAAGGAGGAAGACAGTGATATAGCACAAGACTATACA
GGGGAAGAAAGGCAAGGAAACAGCAACAATTGCCAACCATCATGGAAAGTCTTTCCTGTTCTTGATCA
GGATCTCAATTGTCTTCCTTATGAAGACGACGAGTCTGAGTCACTGGACAATGAGACAGATGTTGAAA
GCTCACCAGGTTTGCTAGCAAAGAAAAAGAGGGCACCCAGTGATCTTGTAGCAAAAATTTCTTTATCA
GATTTGGTCAAGTACTTTGGTATGCCAATTGTAGAAGCATCAAGAAATCTAAATGTTGGGCTCACTGTT
CTCAAGAGAAAGTGCAGAGAATTTGGTATTCCCCGCTGGCCTCATAGGAAGATCAAATCACTTGACAG
TCTCATTCATGATCTTCAGGAAGAGGTAAAGAGCCAAGAATTGGAGGACAGGGAAGCAGCTTTGGCA
GTGGCCAAGAGGCAAAGGATGTTGGAGAGTGAAAAGGAAAACATAGAGAAGAAACCCTTCATGGAC
ATACAAAGTGAGACCAAGAGATTCAGGCAAGATGTTTTCAAGAGAAGACATAGAGCTAGAGCTGTTG
GAAAACACAATTCAACAGTATCCAGCACATAG

>GmRWP-RK1 Protein sequence(326 bp)

MESTLLTNLLVFNNTLQPELMRSVHVYRRGDGEKREVEREFVFSESGSYGEMQATPIPGLVKSRVSEVCEG
HRNGVWLCVFAFHADHTPQFCRIPPVLLVTRNPKLKMIPNLLDDLHVIYKLDRKEEDSDIAQDYTGEERQG
NSNNCQPSWKVFPVLDQDLNCLPYEDDESESLDNETDVESSPGLLAKKKRAPSDLVAKISLSDLVKYFGMP
IVEASRNLNVGLTVLKRKCREFGIPRWPHRKIKSLDSLIHDLQEEVKSQELEDREAALAVAKRQRMLESEKE
NIEKKPFMDIQSETKRFRQDVFKRRHRARAVGKHNSTVSST

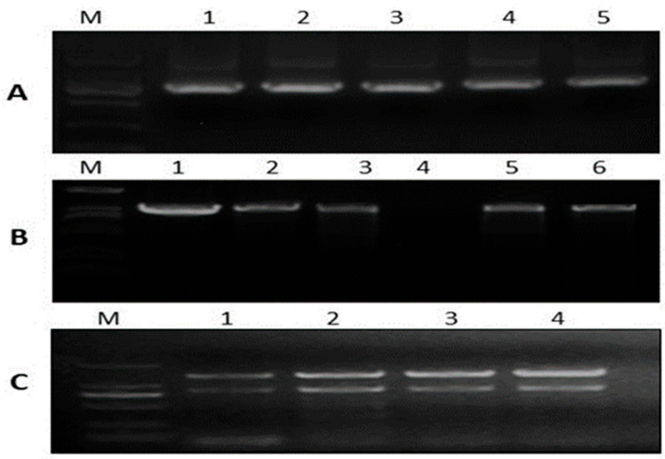

Figure S1. Molecular cloning and confirmation of the recombinant plasmid (T1). (A) Isolation of the full GmRWP-RK1 cDNA and confirmation by PCR, resulting in a 981-bp product. M: Marker (2000 bp); lanes 1–5: Gene amplification bands. (B) Sub-cloning the amplified GmRWP-RK1 product into the pEASY-T1 vector and identifying positive colonies via bacterial PCR with gene-specific primers. (C) After successful Sanger sequencing, dual restriction digestion of the pEASY-T1 vector containing GmRWP-RK1 was performed using BamHI and HindIII enzymes.

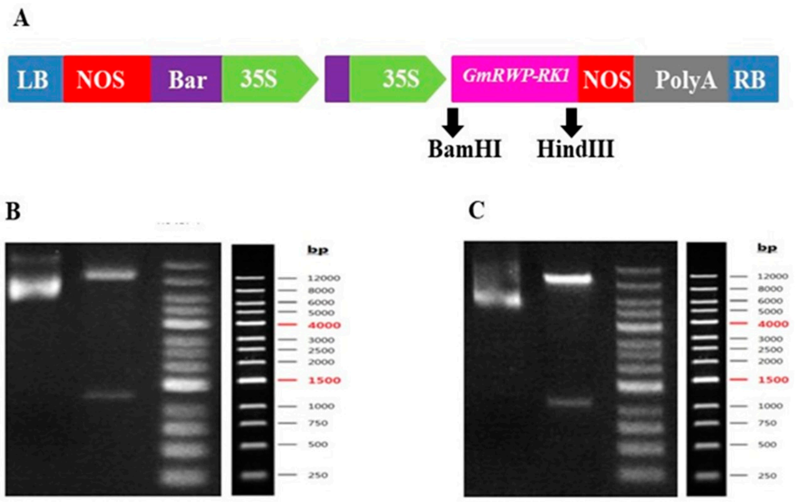

Figure S2. Construction of plant overexpression vector. Schematic illustration of the expression vector. (B) Digestion with BamHI. Lane 1: plasmid, Lane 2: plasmid digested with BamH1 (8820/1229), and Lane 3: DNA Marker. (C) Digestion with Hind111. Lane 1: plasmid, Lane 2: plasmid digested with Hind111(9198/1076), Lane 3: DNA marker.

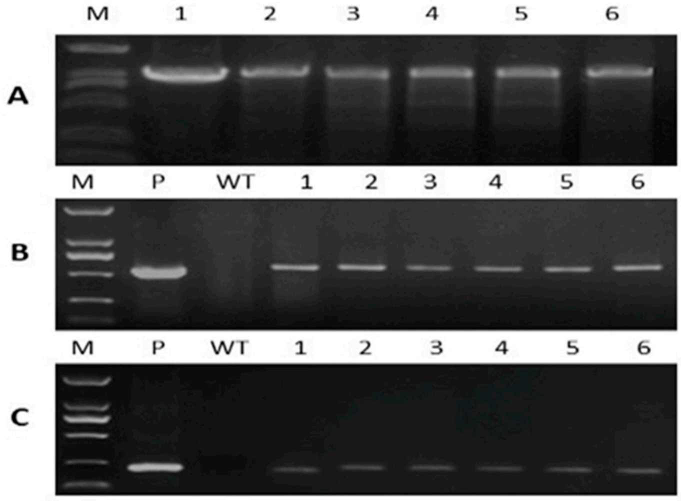

Figure S3. Molecular detection of transgenic plants. (A) Detection of the *GmRWP-RK1* (981-bp) product in transgenic *Arabidopsis* plants. M: DNA marker (2000 kb); lanes 1–6: Transgenic lines. (B) PCR amplification of the Bar gene (selectable marker, 552-bp). WT: Negative control, P: Positive control, lanes 1–6: Transgenic lines. (C) PCR amplification of the NOS terminator gene (127-bp). Lanes 1–6: Presence of the NOS terminator gene in transgenic lines.

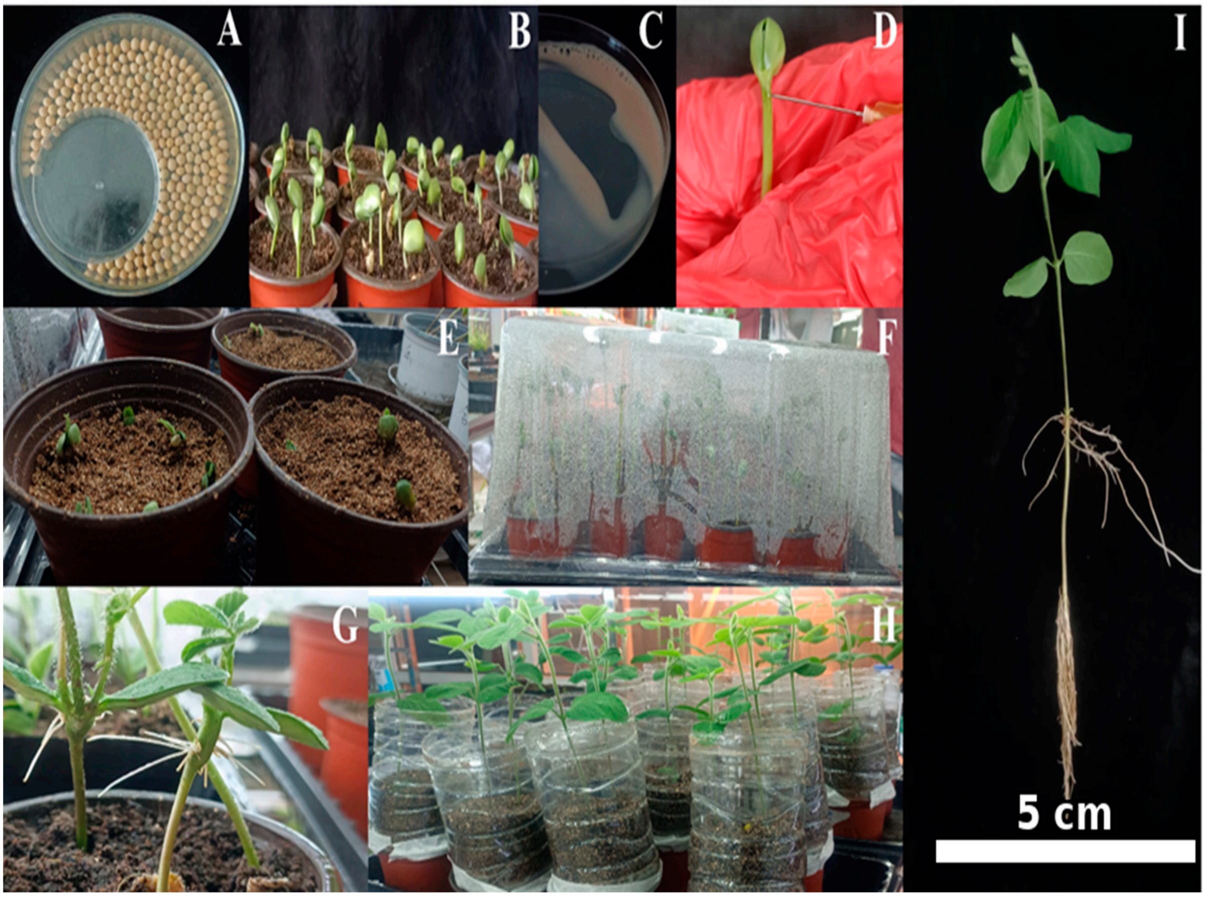

Figure S4. Different stages of the soybean hairy root transformation. (A) Seed sterilization process. (B) The optimal stage for transformation. (C) Inoculation of bacterial paste onto the plant. (D) Injection of bacterial paste into the cotyledonary node and upper hypocotyl. (E) Infected seedlings were transplanted into humidified vermiculite. (F) Infected seedlings transferred to a vent-controlled lid tray for further growth. (G) Appearance of developing hairy roots. (H) After 12 days of inoculation, seedlings were covered with additional humidified vermiculite to promote root regeneration. (I) Transformed soybean plant exhibiting chimeric roots.

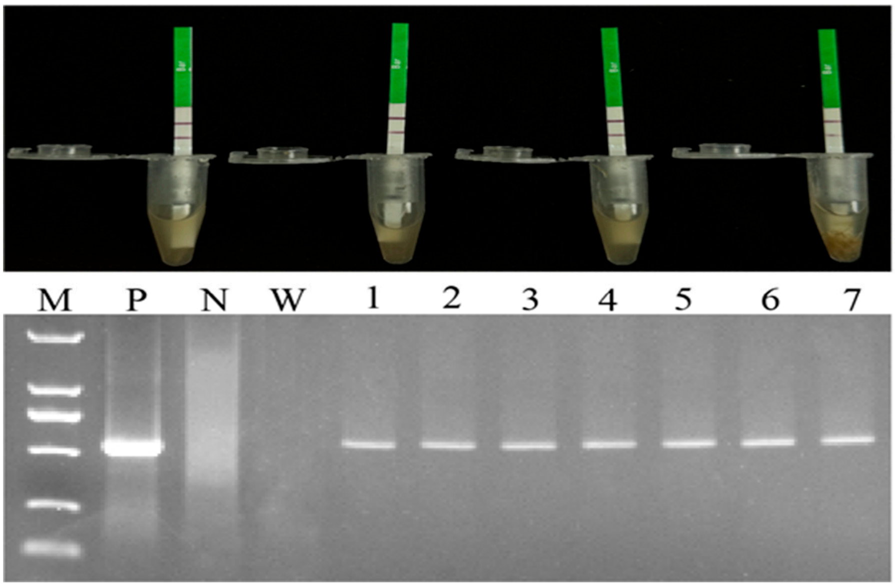

Figure S5. Detection of transgenic hairy roots of soybean. Detection of transgenic hairy roots with Bar rapid strips and PCR amplification of the Bar gene (552-bp) via PCR. Where M= 2000bp, P denoted positive control, and N denoted negative control whereas W denoted Wild type lanes 1 to 7 represent different transgenic samples.

Table S1. List of primers used for PCR implication in transgenic plants.

No.	Gene name	Primer	Primer Sequence (5'to 3')	Bp
**1**	*GmRWP-RK1*	F	ATGGAGTCCACTTTGCTCACCA	21
		R	TGTGCTGGATACTGTTGAATTG	20
**2**	*BAR*	F	TCAAATCTCGGTGACGGGC	20
		R	GTCTGCACCATCGTCAACCACTA	23
**3**	*TNOS*	F	TTATCCTAGTTTGCGCGCTA	20
		R	GAATCCTGTTGCCGGTCTTG	20

Table S2. Primers for the qRT-PCR of *GmRWP-RK1* orthologue's regulatory network in *Arabidopsis*.

No.	Gene name	Primer	Primer Sequence (5’to 3‘)	Bp
**1**	*ATHB22*	F	GGAAGGAGTAAAGGAGTTGGAG	22
		R	TGCACACTTAAGAGCGTCG	19
**2**	*AtTAP46*	F	GGATGGAGAGGGTGGATTTTC	21
		R	TTGTGCCGGTTTAGAGTACTG	21
**3**	*AtASC10*	F	CTCACCTGTCTCATCTCCAAC	21
		R	CTAACTCTTTCAACCCCTCCAC	22
**4**	AtSUMO1	F	CTTTCTTGTTTGATGGGCGTC	21
		R	CCTCCATGTCAAGCTGTAGTG	21
**5**	*AtB120*	F	AGGGAACAAGACGAGGAAATG	21
		R	ACGCTACCTGTTTCATCTGG	20
**6**	*AtSR*	F	ATGTGCTATGAGATACTGAAGGTC	24
		R	GCCCAAATCAACATTACCTCC	21
**7**	*Atgbh1*	F	ACTTTCTCGAAAACACCGGG	20
		R	CTTGAGCACCCCACATATAGG	21
**8**	*AtActin1* (*At3G12110)*	F	ACCGGAATGGTTAAGGCTGG	20
		R	ACTGAGCCTCATCACCAACG	20

Table S3. Primers for the qRT-PCR of *GmRWP-RK1* regulatory network in soybean.

No.	Gene name	Primer	Primer Sequence (5’to 3‘)	Bp
**1**	*GmAP-2 X2*	F	TTCGTGTTTCCGCAATTTGG	20
		R	TCCCTCTCGTCTCTTTCTCTTC	23
**2**	*GmATG-5*	F	CGACAGAACCACATATCCCAG	21
		R	TGCTATAAGGAAAGACGAACCAC	23
**3**	*GmOSM-1*	F	GAGTGTGGCTTTGTGTCTTTG	21
		R	GTGAAAGGCAAATGTAGGGC	20

**4**	*GmOSM-2*	F	CTGCCACCTTTACATTTGCTC	21
		R	CTTAACAATGACCCAAACGGC	21
**5**	*GmEBP-22*	F	AGGAAATGGGACGCAAAGG	19
		R	TGGTCTTCGTTCTGATTCTGG	21
**6**	*GmHIS*	F	CTTTCTCCAGCCCCTTCAC	19
		R	AGGGAACAAAGTGACGAAGAG	21
**7**	*GmActin11*	F	ACCGGAATGGTTAAGGCTGG	20
		R	ACTGAGCCTCATCACCAACG	20

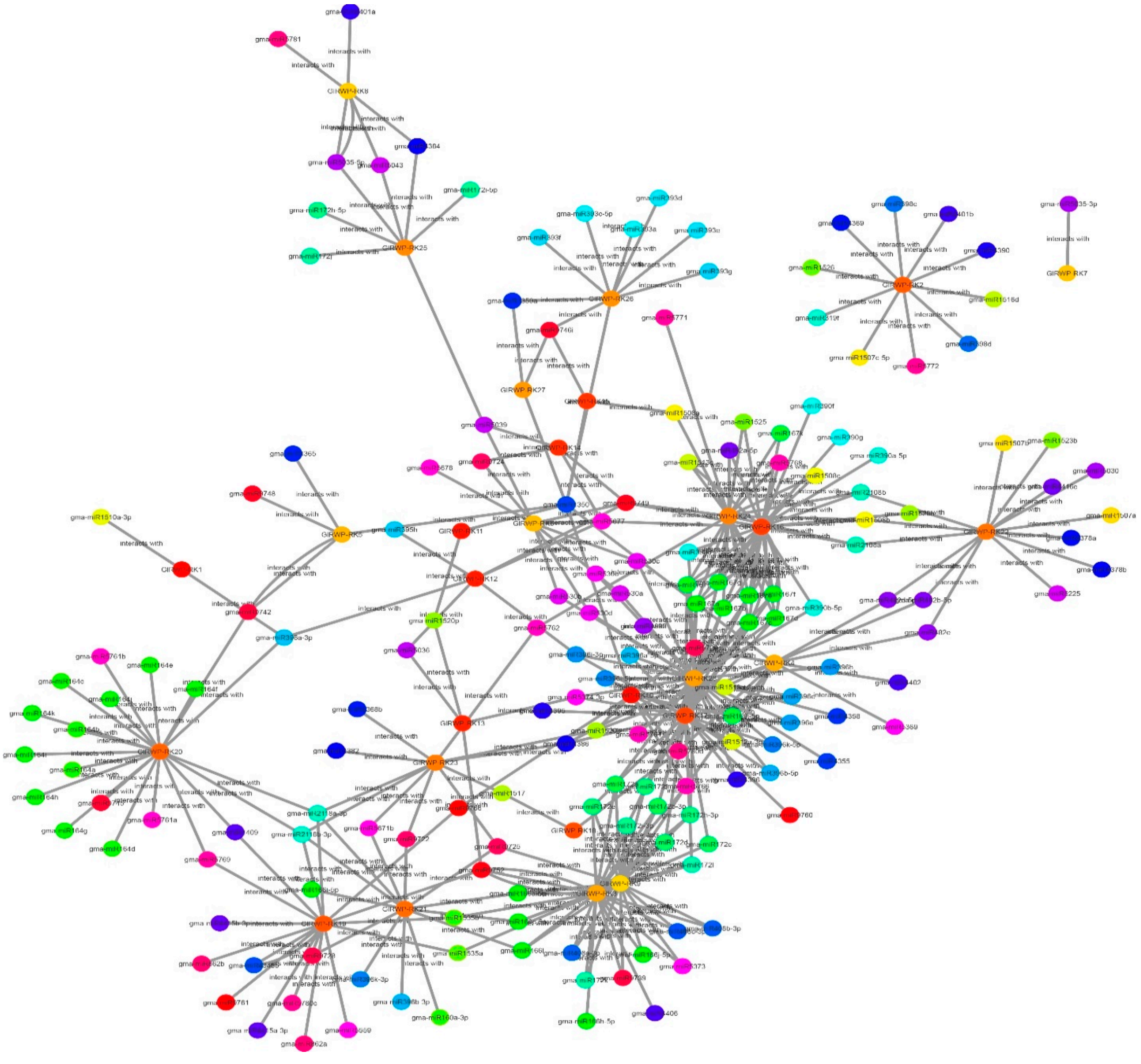

Fig S6. miRNAs prediction network of *GmRWP-RK* transcripts. *GmRWP-RK* transcripts were utilized for the prediction of miRNAs network. Red colors indicate *GmRWP-RK,* and other colors specify different miRNAs network interact with *GmRWP-RK* transcripts.

Table S4. miRNA potential targets in *GmRWP-RK* transcripts. The potential miRNA target sites predicted with the online psRNATarget tool. Expectation Value = 5. (EV – Expectation Value, M – Multiplicity).

**miRNA vs Transcript**	**Alignment**	**E.V**	**Inhibition**	**M**
Gma-miR1510a-3p<br>vs<br>Gm*RWP-RK*1,2	23 UUGUUGUUUUACCUAUUCCACCC 1<br>: : : : : : : : : : : : . . : : : : : : :<br>764 UUCAGGAAGAGGUAAAGAGCCAA 786	5.0	Cleavage	1
gma-miR172a<br>vs<br>Gm*RWP-RK*3	21 AGAAUCUUGAUGAUGCUGCAU 1<br>: . : . . : . . : : . : : : : : : :<br>597 UCUCGGUGUUGUCGAGAUUCU 615	4.0	Cleavage	1
gma-miR482b-5p<br>vs<br>Gm*RWP-RK*4	22 UAUGGGGGGAUUGGGAAGGAAU 1<br>: : . : : . : : : : : : : : : : : . : :<br>174 UUCCUUUUCCAAUCCCCUCUUU 195	4.0	Cleavage	1
gma-miR4350<br>vs<br>Gm*RWP-RK*5,6,7,8	22 UCAAAUGAUUUUGUGUCGUUGG 1<br>: . : : : : . . . : : : : : : : : : : . : :<br>868 AGUAUGACAUUGGAUGAUUUGA 889	5.0	Cleavage	1
gma-miR408b-3p<br>vs<br>Gm*RWP-RK*9	21 AUGCACUGCCUCUUCCCUGGC 1<br>: . . : : : : : : : : . . : : : : : : . .<br>252 GGUGGGGAAGAGAUGGUGGAU 272	5.0	Cleavage	1
gma-miR4350<br>vs<br>Gm*RWP-RK*9	22 UCAAAUGAUUUUGUGUCGUUGG 1<br>: . : . : : . : . : . . : . . : : : : . . :<br>1289 CUCAUCACGCGAGGUUGUUUG 1310	5.0	Cleavage	1
gma-miR1520p<br>vs<br>Gm*RWP-RK*11-16	24 AUGUUGUUAUUGGAUGAUGACGGU 1<br>: : : : : : : . : . : : : . : . : . : . . : . :<br>182 CUUCACAAGAUCCAGUGAUAAUA 205	5.0	Cleavage	1
gma-miR1517<br>vs<br>Gm*RWP-RK*17-18	24 AGUCUUGGUCAAUGUCGUUCGAAA 1<br>: . : : . : : : : : : : : : : : : : : . : :<br>39 AACAGAGAUAUAGCCCAAGACU 418	5.0	Translation	1
gma-miR5559<br>vs<br>Gm*RWP-RK*17-19	21 UACUUGGUGAAUUGUUGGAUC 1<br>: : : . : : . : : : : : : : . : : : : :<br>597 AAUCUCA-AGUUCACCAGGUA 616	4.5	Cleavage	1
gma-miR5769<br>vs<br>GmRWPRK20,21,22	21 UGAGGGAAAUGAAGACGACGA 1<br>. : : . : : : . : : : : . : : : : :<br>208 GUGUUUUCAUUAUUUUCCUCA 288	4.0	Translation	1
gma-miR1508b<br>vs<br>Gm*RWP-RK*23,24	21 UAGAAAGGGGAAUAGCAGUUG 1<br>. : : : : : . : . : : . : : : : : .<br>1973 UUGCUGCUGGUUCCUUUUCUG 1993	4.0	Translation	1

gma-miR5043<br>vs<br>Gm*RWP-RK*25	21 UUAAUGUGUUGUGUUUGUGAG 1<br>: : . : : .: : : : : : : : : : . : :<br>2589 GCCUCAAAUGCAACACAUGGA 2609	4.0	Cleavage	1
gma-miR9746i<br>vs<br>Gm*RWP-RK*26	24 AAAGUGUUUGAAUUUCAAUUAGAU 1<br>: : : : . : : : : : : : : : . : : : : : : : :<br>1142 UAGAUGAAAUUCGAAGACUUC 1165	4.5	Cleavage	1
gma-miR9746i<br>vs<br>Gm*RWP-RK*27	24 AAAGUGUUUGAAUUUCAAUUAGAU 1<br>: : : : . : : : : : : : : . : : : : : : : . :<br>887 CUCUAGAUGAAAUUCGAAGACU 910	4.5	Cleavage	1
gma-miR167b<br>vs<br>Gm*RWP-RK*28	21 UGAAGCUGCCAGCAUGAUCUA 1<br>: : : : : : : : : : : : . : : . : . : . : . : . :<br>250 UAGAGGAUGCUGGUAGUUUA 2521	5.0	Cleavage	1

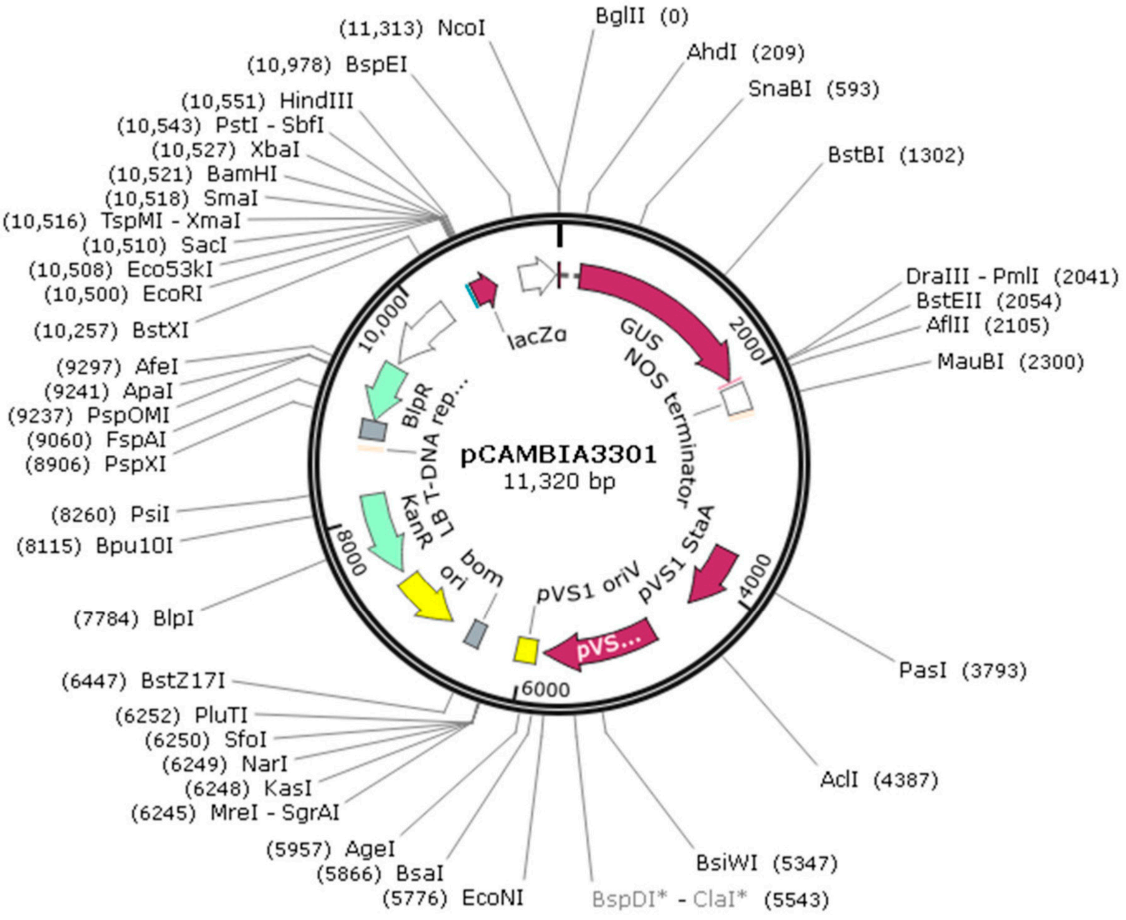

Figure S7. Schematic diagram of the molecular components of the cloning vector. (pCAMBIAI3301 plant overexpression vector).
